# Structural insights into the Caprin-2 HR1 domain in canonical Wnt signaling

**DOI:** 10.1016/j.jbc.2024.107694

**Published:** 2024-08-17

**Authors:** Chun Su, YuCheng Zhong, Zhilei Zhou, Yongtao Li, Yingying Jia, Sichun Xie, Jianfei Zhao, Haofei Miao, Huilian Luo, Zhenyan Li, Zhubin Shi, Lin Li, Xiaomin Song

**Affiliations:** 1 Key Laboratory of Multi-Cell Systems, Shanghai Institute of Biochemistry and Cell Biology, Center for Excellence in Molecular Cell Science, Chinese Academy of Sciences, Shanghai, China; 2 Key Laboratory of Systems Health Science of Zhejiang Province, School of Life Science, Hangzhou Institute for Advanced Study, University of Chinese Academy of Sciences, Hangzhou, China; 3 Westlake Laboratory of Life Sciences and Biomedicine, Hangzhou, China

**Keywords:** canonical Wnt signaling, Caprin-2, HR1 domain, crystal structure, dimerization

## Abstract

The canonical Wnt signaling pathway plays crucial roles in cell fate decisions as well as in pathogenesis of various diseases. Previously, we reported Caprin-2 as a new regulator of canonical Wnt signaling through a mechanism of facilitating LRP5/6 phosphorylation. Here, we resolved the crystal structure of the N-terminal homologous region 1 (HR1) domain of human Caprin-2. HR1 domain is so far only observed in Caprin-2 and its homologous protein Caprin-1, and the function of this domain remains largely mysterious. Here, the structure showed that HR1 domain of human Caprin-2 forms a homo-dimer and exhibits an overall structure roughly resembling the appearance of a pair of scissors. Moreover, we found that residues R200 and R201, which located at a basic cluster within the N-terminal "blades" region, are critical for Caprin-2’s localization to the plasma membrane. In line with this, mutations targeting these two residues decrease Caprin-2's activity in the canonical Wnt signaling. Overall, we characterized a previously unknown "scissors"-like structure of the full-length HR1 domain and revealed its function in mediating Caprin-2’s localization to the plasma membrane.

Canonical Wnt pathway (also known as Wnt/β–catenin pathway) is the best studied Wnt pathway. It plays critical roles in embryonic development as well as multiple aspects of cellular activities, while dysfunction of key components of this pathway is implicated in tumor initiation and progression (1). One striking example is in the colorectal cancers, approximately 80% of which harbor mutations in adenomatous polyposis coli gene, a critical component of the canonical Wnt signaling (2). The seven-pass transmembrane protein frizzled and its cofactor low-density lipoprotein receptor–related protein 5/6 (LRP5/6), when bound by Wnt ligands, will lead to accumulation of the nuclear β-catenin and consequently the activation of Wnt target genes. It is well-accepted that LRP5/6 phosphorylation is a key event during the process of receptor activation. Upon Wnt stimulation, LRP5/6 forms complex with frizzled, disheveled, axin, casein kinase 1, and glycogen synthase kinase 3 (this complex is also known as LRP5/6 signalosome), in which LRP5/6 is phosphorylated and thus activated (3). During this process of LRP5/6 activation, disheveled is believed to play a critical role probably through mediating LRP5/6 aggregation, although the exact mechanism underlying this remains largely unclear.

Caprin family proteins are initially identified as cytoplasmic activation/proliferation associated proteins (4). Human Caprin family comprises two members, Caprin-1 and Caprin-2. These two members share two homologous domains, homology regions 1 and 2 (HR1 and HR2); while, Caprin-2 contains an extra C-terminal C1q domain which is absent in Caprin-1. Researches so far indicated that Caprin-1 and Caprin-2 are functionally nonredundant. Early studies indicate that Caprin-1 plays a role in cellular proliferation, and suppression of Caprin-1 expression decreases the cell proliferation rate (4, 5). Later, most findings have related Caprin-1 to the formation of RNA granules including stress and neuronal granules (6, 7, 8, 9). Besides, Caprin-1 has also been reported to play a role in viral infection and tumorigenesis (10, 11, 12). Compared to Caprin-1, the function of Caprin-2 is much less clear. One report related Caprin-2 to the developmental transition from rapid proliferation towards terminal differentiation (13). Caprin-2 has also been reported to be involved in the fibroblast growth factor-regulated lens fiber cell differentiation (14, 15). Similar to Caprin-1, Caprin-2 can also bind to mRNA, but Caprin-2 seems to form different RNA granules from the ones by Caprin-1; and the effects caused by knockdown of Caprin-1 could not be rescued by Caprin-2 and *vice versa* (8).

Previously, we revealed that Caprin-2, but not Caprin-1, binds directly to LRP5/6, which facilitates both Wnt-induced and Wnt-independent LRP5/6 phosphorylation (16, 17). We also resolved the crystal structure of the C-terminal C1q domain of Caprin-2 (Cap2_CRD) (18). Cap2_CRD exhibits a trimeric assembly with the typical jelly roll structure generally observed in C1q family and also in tumor**-**necrosis factor family proteins. Our studies suggested that trimerization of CRD is required for Caprin-2's function in promoting LRP5/6 phosphorylation probably through contributing to the aggregation of LRP5/6 (18). Part of the Caprin-2 HR1 domain (Cap2_HR1), roughly corresponding to the "bow" part of the HR1 "scissors" structure, has previously been resolved by Wu *et al.*, and they suggested that this region might be the sole one in the HR1 domain that could be well purified and crystalized (19). However, though limited trypsinization combined with mass spectrometry (MS) and N-terminal protein sequencing, we discovered a compact and stable region covering the whole HR1 domain, which could be crystallized with decent diffraction resolution. HR1 domain is so far only observed in Caprin-1 and Caprin-2, and this domain shares no sequence similarities with any other known motifs, indicating that HR1 may perform a unique function. In this study, we determined the crystal structures of Cap2_HR1 *via* selenium single-wavelength anomalous diffraction method. The structure of Cap2_HR1 adopts a unique dimeric "scissors"-like assembly that could be divided into two subdomains—the N-terminal lobe (N-lobe) that corresponds to the "blades" and the C-terminal lobe (C-lobe) that corresponds to the "bow." Within a coiled coil region of the N-lobe, there is a basic cluster, which has previously been implicated in a role of RNA binding (8). Surprisingly, we found that Cap2_HR1 binds specifically to phosphatidylinositol 4-phosphate (PI4P), and mutations targeting R200 and R201 in this basic cluster not only disrupt Caprin-2's association with PI4P but also lead to altered cellular localization as well as decreased activities of Caprin-2 in canonical Wnt signaling.

## Results

### Overall structure of Cap2_HR1

**Figure S1:**
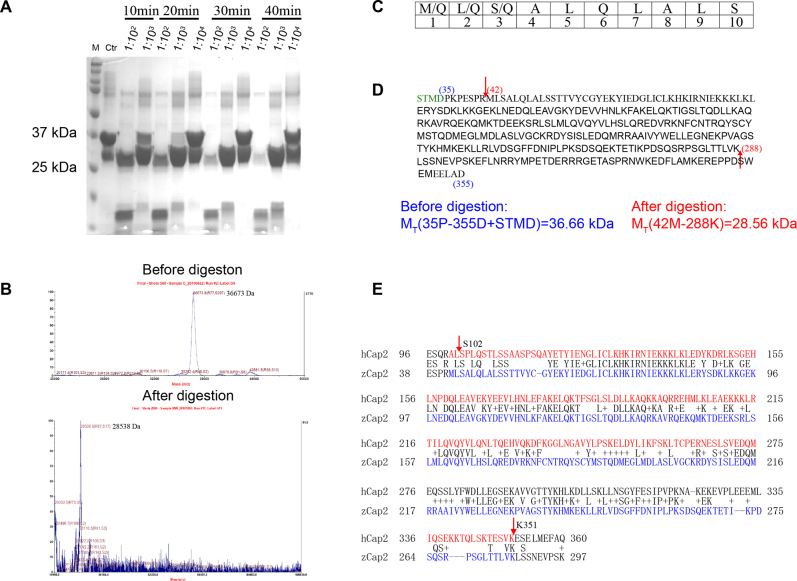


Our initial attempt to crystallize zebrafish Cap2_HR1 (aa S35-D355) failed. To remove possible flexible regions interfering with crystallization, we subjected purified zCapr2_HR1 protein samples to limited trypsinization. The sequence of the proteolyzed product was determined by a combination of N-terminal amino acid sequencing and MS, which was revealed to encompass aa M42 to K288 (Fig. S1, *A*–*D*). The purified zCap2_HR1 (aa M42–K288) was then crystallized, which however showed very poor diffraction abilities of ∼8 Å. We then tried the corresponding region of hCap2_HR1 (aa S102–K351) based on sequence alignment between hCaprin-2 and zCaprin-2 (Fig. S1*E*), and this hCap2_HR1 protein was crystallized with significantly improved diffractions. Structure determination using selenium single-wavelength anomalous diffraction/selenium multi-wavelength anomalous diffraction datasets collected from the wild-type (WT) hCap_HR1 crystals failed probably due to the insufficient phasing power by the intrinsic three methionine residues in the hCap_HR1 sequence. To increase the anomalous signal of selenium, we introduced three more methionine residues (I217M/L251M/L301M) to hCap2_HR1 (we selected the three sites based on sequence alignments, namely these three residues in hCaprin-2 are replaced by methionine residues in either zebrafish or mouse Caprin-2). The crystals of this triple mutant I217M/L251M/L301M were grown under similar conditions to those of WT hCap2_HR1. Datasets collected from I217M/L251M/L301M crystals allowed for successful structure determination by using selenium multi-wavelength anomalous diffraction method with a resolution to 2.32 Å.

**Figure 1:**
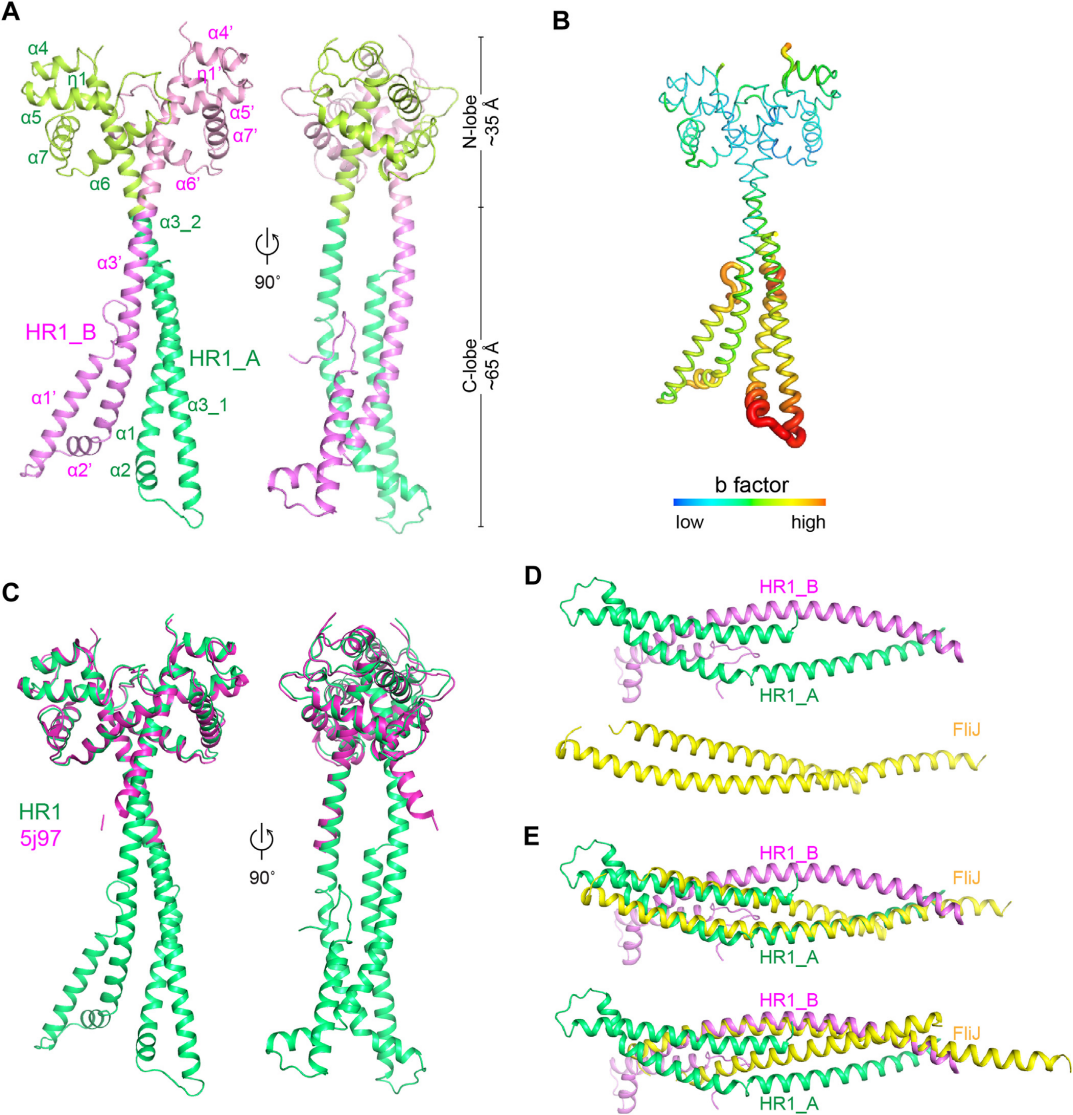
**Structures of hCap2_HR1.***A*, two copies of hCap2_HR1 forms a dimer with the overall structure roughly resembling the shape of a pair of "scissors," which could be divided into two subdomains: a "blade"-like N-lobe and a "bow"-like C-lobe. *B*, the N-lobe of hCap2_HR1, when compared to the C-lobe, exhibits higher B factors, especially in its antiparallel helix-loop-helix region. *C*, part of the human Caprin-2 HR1 domain has been previously resolved by Wu *et al.* (PDB code: 5J97), which roughly corresponds to the "bow" part of hCap2_HR1. *D*, the most homologous protein three dimensionally for the N-lobe of hCap2_HR1 is flagellar FliJ protein (PDB code: 3ajw). FliJ forms an anti-parallel, α-helical coiled-coil structure. *E*, superimposition of FliJ with hCap2_HR1 chain A or chain B shows a limited similarity between these two structures. Images of structures were generated using PyMOL (https://pymol.org/). HR, homologous region.

Two copies of the Cap2_HR1 were found in an asymmetric unit and assigned as chains A and B (Fig. 1*A*). AA S102-A111 and P320-K351 in chain A and S102-A117 and K321-K351 in chain B are disordered. The HR1 domain was initially defined by Grill *et al.* as a conserved region in Caprin-1 and Caprin-2, which encompasses amino acids N124-S315 in human Caprin-2 (4). Therefore, the ordered structure we observed here covers the full-length HR1 domain. As shown in Figure 1*A*, two molecules of hCap2_HR1 form an asymmetric dimer, with the overall structure roughly resembling the shape of a pair of "scissors" measuring ∼100 Å in height and ∼60 Å in width. The structure of hCap2_HR1 could be divided into two subdomains: a "blade"-like N-lobe (aa A112-L205) and a "bow"-like C-lobe (aa K206-P320). The blades of the "scissors" consist of four α-helices in chain A and three α-helices in chain B. The four α-helices of chain A are helix α1 (aa S113–L150), short helix α2 (aa P158–E165), helix α3_1 (aa Y167–G186), and part of helix α3_2 (aa S188–L205). While, the N-terminal region (aa Y118–L129) in the N-lobe of chain B melts to a short helix-loop-helix structure instead of α helix such as in chain A, and the remaining part of chain B is constituted of helix α1’ (aa K130–L150), helix α2’ (aa P158–E165), and part of helix α3’ (aa E168–L205). The "bows" of the "scissors" exhibits a symmetric dimeric assembly, with each subunit composed of six helices including five α helices (α3–α7) and one 3_10_ helix (η1) in the C termini of each chain. The parts of helices α3-2 and α3′ in the C-lobe of chains A and B, respectively, packed together forming a parallel coiled-coil dimer with the rest helices in the N-lobe forming two antiparallel dimers. Of note, compared to the C-lobe, the N-lobe, especially its antiparallel helix-loop-helix region, exhibited higher B-factors (Fig. 1*B*), indicating a greater flexibility of this region.

Wu *et al.* previously solved part of the HR1 domain of human Caprin-2 (PDB code: 5J97), which encompassing aa 199 to 320 (19). Superimposition of this structure and hCap2_HR1 gave rise to an RMSD of 0.3669 Å for 114 Cα (Fig. 1*D*). Search for other similar structures for the full-length hCap2_HR1 using Dali and secondary-structure matching server revealed no closely related structures. When single N-lobe or C-lobe of hCap2_HR1 is used for the search, a couple of structures with low sequence similarity (generally less than 10%) were identified, although these structures could be superimposed only partially with that of the N-lobe or the C-lobe of hCap2_HR1. According to the Z-score calculated by Dali sever, the most homologous protein three dimensionally for the N-lobe of HR1 is flagellar FliJ protein (PDB code: 3ajw) (20), a soluble component of type III protein secretion system (Fig. 1, *D* and *E*). FliJ shows a long monomeric α-helical structure, which is bent roughly at the middle of the long helix, thus forming an antiparallel, α-helical coiled-coil structure. FliJ uses different regions along the helix to interact with different partners including FlgN, FliT, FliH, FliT, and FliH (20). Considering the quite flexibility of the N-lobe of hCap2_HR1, it is reasonable to surmise that Cap2_HR1 might adjust the conformations and utilize different regions along the α-helix to accommodate distinct partners.

### Dimeric interface of Cap2_HR1

**Figure 2:**
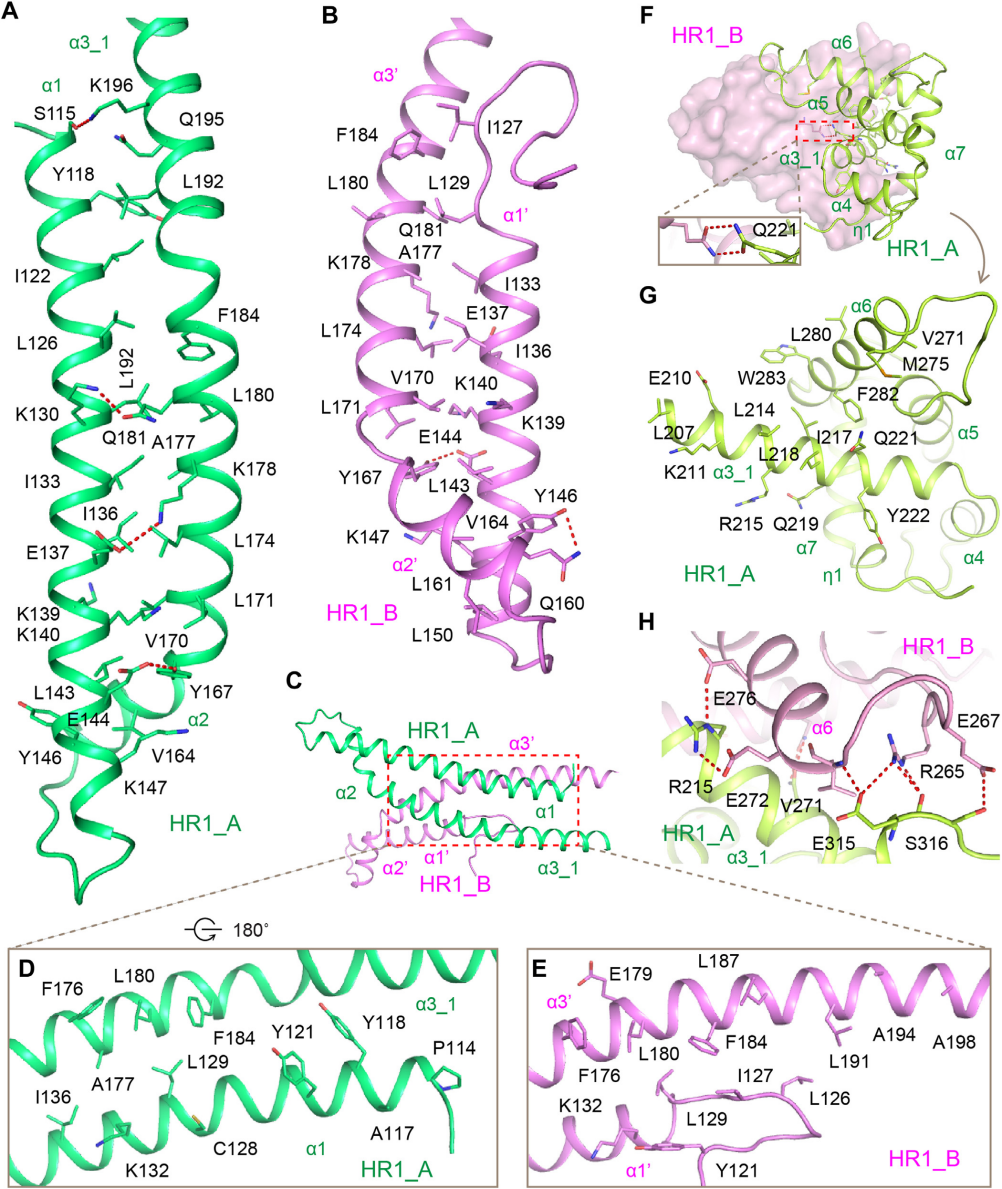
**Details of the interface of hCap2_HR1 trimer.***A* and *B*, in the N-lobe, helices α1 and α1′ fold back toward and form intramolecular interaction with helices α2, α3-1, and α3-2 in chain A and helices α2′ and α3′ in chain B, respectively, thereby forming two anti-parallel coiled coils. *C*–*E*, details of the intermolecular interactions between the two N-lobes. *F*, *G*, and *H*, details of the intermolecular interactions between the two C-lobes. Images of structures were generated using PyMOL. HR, homologous region.

Both of the N- and C-lobes are involved in the formation of Cap2_HR1 homodimer. The dimeric interface has a total buried area of 2414.4 Å^2^. In the N-lobe, helices α1 and α1′ fold back toward and form intramolecular interaction with helices α2, α3-1, and α3-2 in chain A and helices α2′ and α3′ in chain B, respectively, whereby forming two anti-parallel coiled coils (Fig. 2, *A* and *B*). Residues I133, I136, E137, K139, K140, L143, E144, Y146, K147, V164, Y167, V170, L171, L174, A177, K178, L180, Q181, and F184 in both of two chains are involved in coiled coil formation *via* hydrophobic interaction. Residues Y118, I122, L126, L192, and Q195 in chain A, and I127, L129, L150, Q160, and L161 in chain B also contribute to the assembly of each coiled coil. Additionally, residues S115, K130, E137, E144, and Y146 form hydrogen bonds with residue K196, Q181, K178, Y167, and Q160, respectively, in chain A or/and B, further stabilizing the coiled-coil structure.

The intermolecular interaction between the two N-lobes is achieved by the N-terminal loop of chain B, and the helices α1, α1′, α3-1, α3-2, and α3′ of chain A or B (Fig. 2, *C*–*E*). In detail, residues Y121, L129, K132, H172D, F176, L180, and F184 form both chains, residues P114, A117, Y118, C128, and I136 in chain A, and L126, I127, L187, L191, A194, and A198 in chain B constitute a hydrophobic core. Besides, residue K132 in chain A interacts with E179 in chain B *via* salt bridges. The hydroxyl groups of residues Y118 and Y121 of chain A contact the main chains of L126 and Y127 of chain B *via* hydrogen bonds. The two C-lobes from chains A and B are symmetric (Fig. 2*F*). Helices α3_2, α3′, α6, and α6′ hold two C-lobes together. The C-terminal parts of helices α3_2 and α3′ intertwine with each other in the core region of C-lobe dimer. It is worth noting that two Q221 residues from chains A and B form two hydrogen bonds in the core region (Fig. 2*F*). Residues L207, E210, K211, L214, R215, I217, L218, Q219, and Y222 in helices α3_2 and α3′, and V271, M275, L280, F282, and W283 in helices α6 and α6′ form a hydrophobic core to maintain the dimeric fold of HR1 C-lobe (Fig. 2*G*). In the surface of hCap2_HR1 C-lobe dimer, the side chains of R215 (in helix α3), E315 and S316 (in helix α8), and the main chain of E315 from one molecule, form extensive hydrogen bonds with the side chains of R265 and E267 (in α5/α6 loop), E272 and E276 (in helix α6), and the main chain of V271 from the other molecule (Fig. 2*H*). These hydrogen–bonding interactions further stabilize the dimerization of HR1 C-lobe.

**Figure S2:**
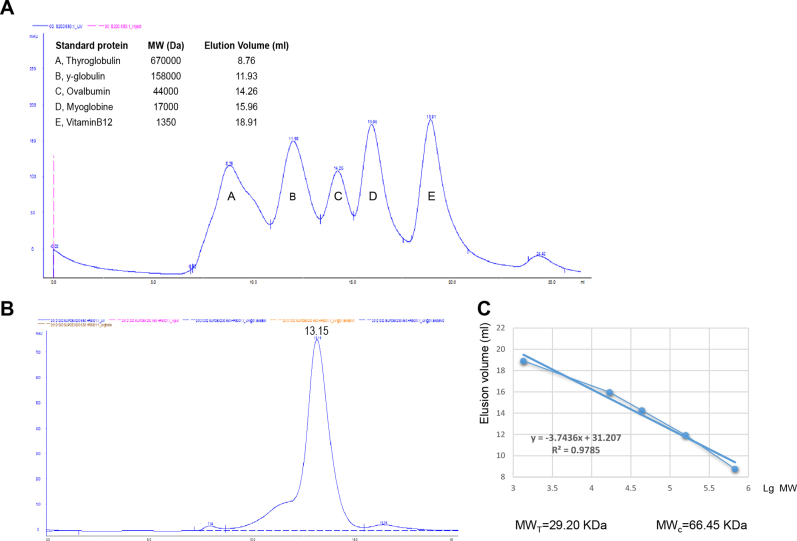


Our structural analysis above suggested that Cap2_HR1 forms a dimer, which is stabilized by an extensive network of hydrogen bonds and hydrophobic interactions. To test whether Cap2_HR1 might exist as a dimer in solution, we evaluated the molecular weight (MW) of Cap2_HR1 in solution by using gel filtration chromatography. We generated a calibration curve on the size-exclusion column Superdex 200 and then used it to estimate the MW of Cap2_HR1 in solution being 66.45 kDa (Fig. S2). Considering the theoretical MW of Cap2_HR1 being 29.2 kDa, we tend to conclude that Cap2_HR exists as a dimer in solution.

### R200 and R201 are critical for Caprin-2’s function in Wnt signaling

**Figure 3:**
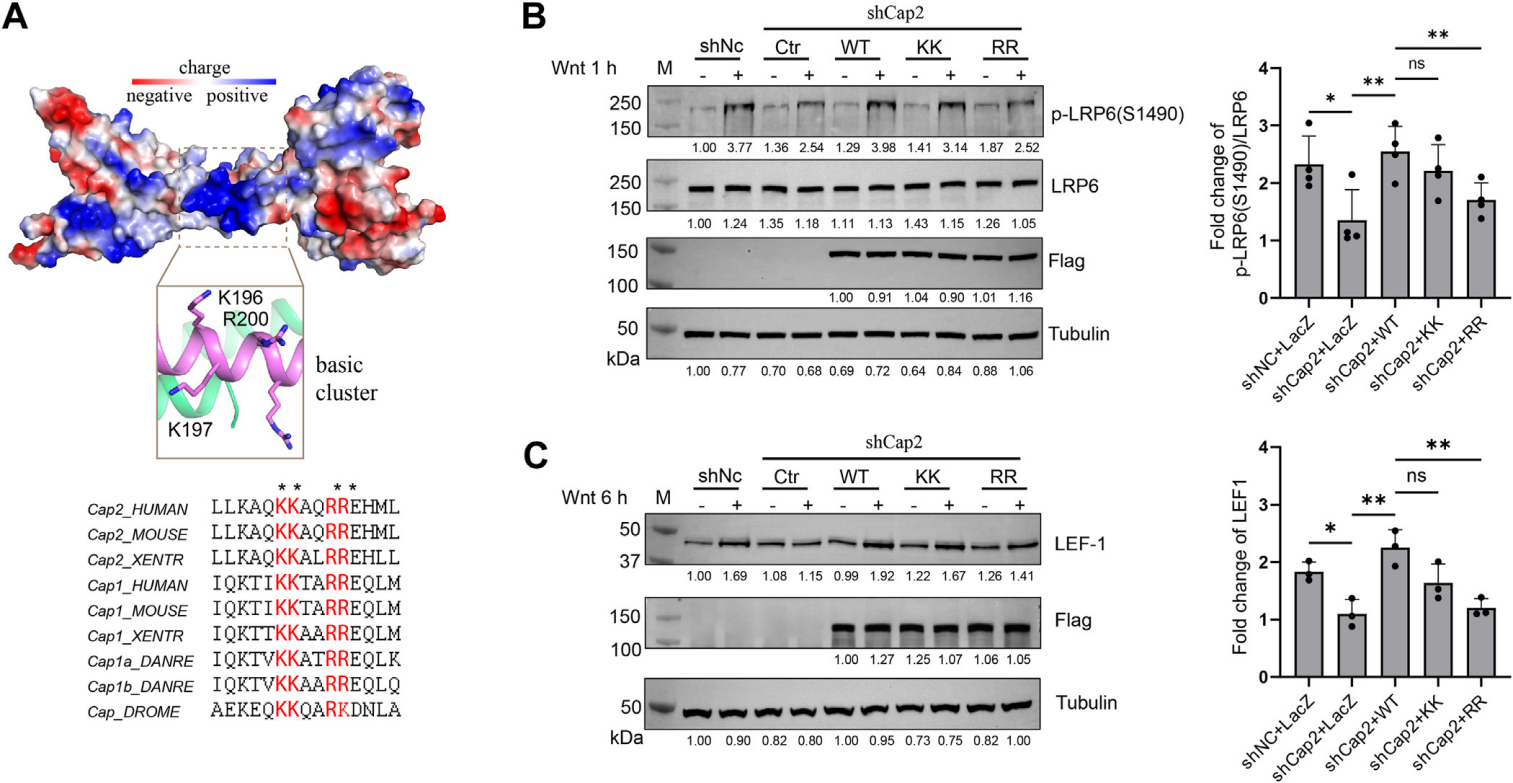
**R200/R201 are critical for Caprin-2’s function in Wnt signaling.***A*, surface view of the basic region in the N-lobe of hCap2_HR1. Basic residues K196, K197, R200, and R201, which are located at the core of the basic region, are evolutionarily conserved and solvent-exposed. Images of structures were generated using PyMOL. *B* and *C*, knocked down rescue assay to examine whether residues K196/K197 and R200/R201 might affect Caprin-2’s activity in facilitating LRP6 phosphorylation (*B*) and Wnt target gene *LEF1* expression (*C*). U2OS cells were infected by lentivirus encoding the shRNA-targeting Caprin-2 or by lentivirus encoding shRNA control for 48 h. Mixture of the target plasmid (Caprin-2 WT, R200E/R201E, or K196E/K197E), P3000 and Lipo-3000 in Opti-minimal essential medium was added to cells for 24 h. Cells were then treated with control CM or Wnt-3a CM for 1 h (B) or 6 h (C) and were then collected for Western blot analyses with the indicated antibodies. Phosphorylated LRP6 and total LRP6 levels were normalized to tubulin and then their ratios were calculated. LEF1 levels were normalized to tubulin. The knockdown efficiency of Caprin-2 was checked by quantitative PCR (Fig. S2). Wnt-3a CM and control CM were prepared as we described previously (33). Data are representative of three independent experiments. The immunoblots are quantified by ImageJ software (https://imagej.net/ij/download.html) (34) and values are given beneath each band. A two-tailed *t* test was used for statistical analysis by using GraphPad (https://www.graphpad.com/). ∗*p* ≤ 0.05, ∗∗*p* ≤ 0.01, and ∗∗∗*p* ≤ 0.001. CM, conditioned medium; HR, homologous region; LRP, low-density lipoprotein receptor–related protein.

**Figure S3:**
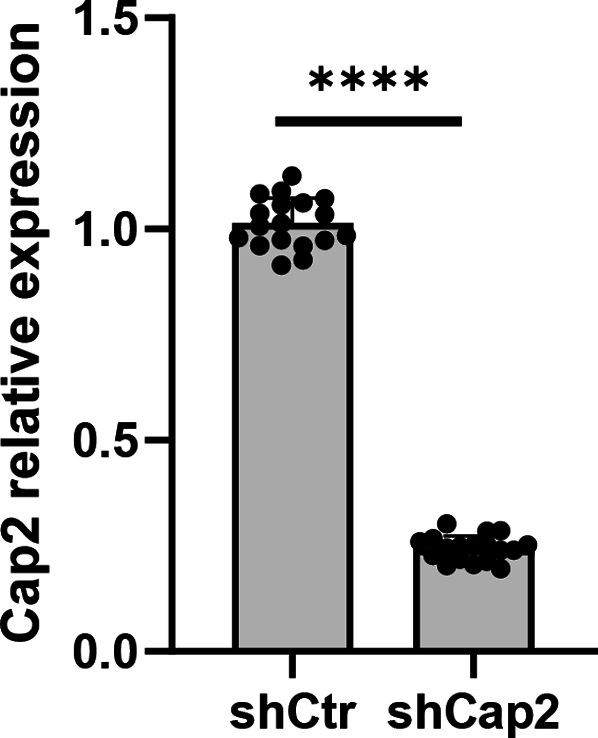


Close to the junction of the N-lobe and the C-lobe, a cluster of basic residues aligned along the solve-exposed side of the coiled coil of the N-lobe (Fig. 3*A*). Primary sequence analysis with PredictNLS and Predictprotein indicated that this cluster includes a potential nuclear localization signal and is predicted to have a capability of binding nucleotides. Several studies have confirmed that the HR1 domain of Caprin-2 and Caprin-1 can bind to RNA *in vitro* (6, 8). So far, the *in vivo* function of the HR1 domain remains largely unclear. To investigate whether this basic cluster might affect Caprin-2’s activity in Wnt signaling, we constructed two double mutants, replacing K196/K197 or R200/R201—which are exposed to the solvent region and evolutionarily conserved—with glutamate residues (Fig. 3*A*). We next carried out knocked down-rescue assay in U2OS cells to examine whether K196/K197 or R200/R201 might affect Caprin-2’s activity in facilitating Wnt-induced LRP6 phosphorylation. As shown in Figure 3*B*, compared with WT Caprin-2, R200E/R201E led to a significantly reduced Wnt-induced LRP6 phosphorylation and K196E/K197E showed a minor effect without a statistically significance. We next examined effects of these two double mutants on Wnt target gene LEF1 expression. Consistent with their effects on Wnt-induced LRP6 phosphorylation, R200E/R201E significantly reduced Caprin2’s activity in promoting LEF1 expression, while K196E/K197E showing a minor effect that was not statistically significant (Fig. 3*C*). The knockdown efficiency of Caprin-2 was confirmed by quantitative PCR (qPCR) (Fig. S3). Together, these data suggested that residues R200 and R201 could be critical for Caprin-2 to function in Wnt signaling.

### R200 and R201 mediate Caprin-2’s localization to the membrane

**Figure 4:**
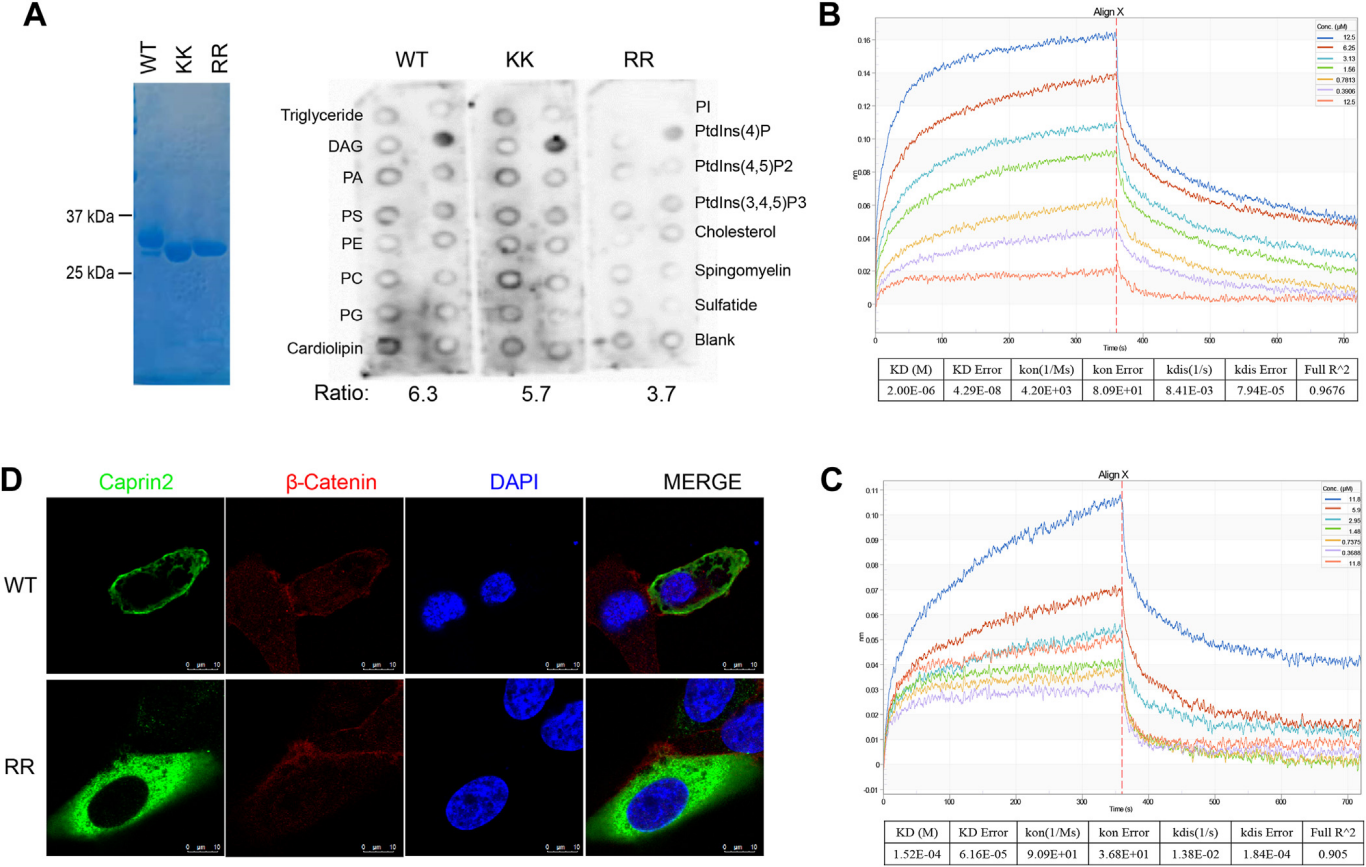
**R200/R201 mediate Caprin-2’s localization to the membrane.***A*, membrane lipid strips probed with purified 6×His tagged hCap2_HR1 WT, K196E/K197E, or R200E/R201E, respectively. Relative protein levels were quantified using ImageJ software (34). *B* and *C*, measurement of the binding affinity of PI4P with hCap2_HR1 WT (*B*) or with the R200E/R201E double mutant (*C*). Biotinylated PI4P were loaded onto streptavidin sensors and were then incubated with different concentrations of purified hCap2_HR1 WT (*B*) or R200E/R201E (*C*). Curves are shown in *colors* that are corresponding to sample concentrations as shown in the *top right-hand corner*. K_dis_ (dissociation rate) and K_on_ (on-rate) as well as the corresponding errors were calculated with Octet Data Analysis 11.0. *D*, subcellular localization analysis of Caprin-2 WT and R200E/R201E mutant. Endogenous β-catenin was used as an indicator of the plasma membrane. Flag-tagged full-length Caprin-2 WT or R200E/R201E mutant was transfected into U2OS cells grown on glass coverslips. After being fixed with 4% (w/v) paraformaldehyde and permeabilized with 0.1% (v/v) Triton X-100, anti-Flag, or anti-β-catenin was added followed by secondary antibody treatments. Photos were taken with Leica TCS SP8. Panels (*left to right*): Caprin2 (*green*); β-catenin (*red*); 4′, 6-diamidino-2-phenylindole (*blue*); merged images. Scale bars represent1 0 μm. HR, homologous region; PI4P, phosphatidylinositol 4-phosphate.

So far, we found that R200 and R201 are the two residues that could be critical for Caprin-2’s function in Wnt signaling. Solvent-exposed basic amino acid residues like lysine and arginine could function in several ways, such as binding to nucleic acid phosphates in RNA/DNA, binding to acidic lipids in membranes, and interacting with acidic residues in other proteins. Our previous results demonstrated that Caprin-2 binds to LRP5/6 and facilitates its phosphorylation, which is an event that occurs close to the plasma membrane (16, 17). Therefore, it is reasonable to surmise that R200 and R201 might bind to acidic lipids in the plasma membrane. To test this possibility, we purified recombinant 6 × His-tagged hCap2_HR1 WT and the two double mutants K196E/K197E and R200E/R201E and then carried out *in vitro* lipid binding assay using the Membrane Lipid Strips (Echelon Bioscience). As shown in Figure 4*A*, WT Cap2_HR1 exhibited a specific interaction with PI4P; while K196E/K197E led to a slight, and R200E/R201E led to a more apparent decrease of this interaction. This result indicated that hCap2_HR1 binds specifically to PI4P, in which R200 and R201 might play a critical role. To further validate this result, we assessed whether the double mutant R200E/R201E could affect the interaction of hCap2_HR1 with PT4P by performing the biolayer interferometry assay. As shown in Figure 4, *B* and *C*, WT hCap2_HR1 exhibited a binding affinity with PI4P (K_D_ = 2.0 μm), which is about 70-fold higher than that of the double mutant R200E/R201E (K_D_ = 152 μm). This result further confirmed that R200 and R201 played a critical role in hCap2_HR1’s interaction with PI4P. PI4P is one of the key lipids that maintain electrostatic properties of the plasma membrane (21, 22, 23). Therefore, we then examined whether R200E and R201E might affect subcellular localization of Caprin-2. We transfected the Flag-tagged full-length Caprin-2 WT and the R200E/R201E double mutant into U2OS cells. We found that Caprin-2 WT were mostly localized to the plasma membrane; by contrast, the double mutant R200E/R201E were distributed throughout the cytoplasm (Fig. 4*D*). Overall, these results indicated that residues R200 and R201 are critical for Caprin-2’s membrane localization probably through binding to PI4P in plasma membrane.

## Discussion

We previously identified Caprin-2 as a positive regulator of canonical Wnt signaling and we found that Caprin-2 could promote LRP6-Axin-glycogen synthase kinase 3 and LRP6–CDK14–cyclin Y complex, which mediates LRP6 phosphorylation in Wnt-on and Wnt-off state respectively (16, 17). We also resolved the structure of the C1q domain of Caprin-2 (Cap2_CRD), which forms a homo-trimer, and mutations disrupting this trimerization showed a decreased capacity in facilitating LRP5/6 phosphorylation without affecting Caprin-2–LRP5/6 interaction (18). These findings indicated that Caprin-2 might serve as a scaffold protein, which plays a role in LRP5/6 aggregation and assembly of different LRP5/6 signalosomes. In this study, we determined the crystal structure of Cap2_HR1 (Fig. 1*A*). We observed that the anti-parallel helix-loop-helix structure exhibits a high value of temperature factor (Fig. 1*B*), indicating that this region in the N-lobe has considerable conformational flexibility, which might mediate Caprin-2’s interactions with different partners to regulate LRP5/6 activation.

Several studies have related Caprin family proteins to RNA-related functions. The HR1 domain of Caprin-2 contains a basic residue-rich region, which is located at the solvent-exposed side of the N-lobe α-helix. According to sequence prediction, this region is expected to play a role in binding RNA as well as leading the protein to the nucleus. Indeed, *in vitro* binding assays showed a binding of the HR1 domain of both Caprin-1 and Caprin-2 with RNA (6, 8). However, according to our results, this cluster has an unexpected function in mediating localization of Caprin-2 into the plasma membrane. Our previous findings (Caprin-2 regulates phosphorylation of LRP5/6 that is a membrane co-receptor) and our observations in this study (Caprin-2 could be located to the membrane and interacts with PI4P) support a function of Caprin-2 happening close to the plasma membrane. At this stage, we could not rule out the possibility that Caprin-2 might also function in Wnt signaling through other mechanisms that involve interactions with RNAs. Whether Caprin-2 binds RNAs to regulate Wnt signaling and if so, how the HR1 domain is involved in bindings with different partners warrant further studies.

On the other hand, our results showed that Cap2_HR1 binds directly with PI4P mainly through R200 and R201 (Fig. 4, *A*–*C*), and mutation of these two residues disrupt Caprin-2’s localization into the plasma membrane as well as decrease Caprin-2’s activity in canonical Wnt signaling (Fig. 3, *B*–*E* and Fig. 4*D*). Based on these results, we surmised that Caprin-2 might localize into the membrane directly through its interaction with membrane PI4P. On the other hand, we observed an intracellular interaction within Caprin-2, which involves its N-terminal HR1 region (data not shown). Therefore, another possibility is that the binding of Caprin-2 with PI4P may induce a conformational change of Caprin-2, which facilitates Caprin-2’s interaction with LRP6 or other components in the LRP6 signalosome, consequently leading to Caprin-2’s recruitment into the plasma membrane. Of note, PI4P is a precursor of phosphatidylinositol 4,5-bisphosphate (PIP2). Increasing evidence has pointed out the important role of PIP2, also a phospholipid component of cell membranes, in the activation of LRP5/6 (21, 23). PIP2 is synthesized majorly through phosphorylation of phospatidylinositol by phosphatidylinositol 4-kinases, followed by further phosphorylation of PI4P by phosphatidylinositol 4,5-biphosphate kinases (24). The fact that membrane phospholipid PIP2 is involved in LRP5/6 activation indicates multiple mechanisms may coexist to form a complex regulatory network for a prober activation of LRP5/6. Whether binding of Caprin-2 with PI4P may play a role in PIP2-mediated LRP6 phosphorylation remains unknown. Further researches are required to delineate how the interaction between Caprin-2 and PIP4 functions in membrane localization of Caprin-2 and whether this interaction may affect the activity of PIP2 in promoting LRP5/6 phosphorylation.

## Experimental procedures

### Protein expression, purification, and mutagenesis

The HR1 domain of human and zebrafish Caprin-2 were cloned from the full-length human and zebrafish Caprin-2, respectively, which were conducted as described before (16). The initial attempt to crystallize zebrafish Cap_HR1 (zCap_HR1) encompassing aa S35-D355 failed. Through limited trypsinization combined with N-terminal amino acid sequencing and MS, a new fragment of zCap_HR1 encompassing aa M42-K288 was purified and crystallized. Due to a poor diffraction ability of zCap_HR1, the corresponding fragment of human Cap2_HR1 (aa S102-K351) was designed and inserted into a modified PET-28a vector with the thrombin cleavage site replaced by a tobacco etch virus (TEV) protease cleavage site. After verified by DNA sequencing, the plasmid was transformed into BL21-CodonPlus-RIL (Agilent Technologies, 230240), which provides extra copies of the argU, ileY, and leuW tRNA genes. Protein expression was induced in LB medium at 20 °C for ∼22 h after *A*_600_ arrived ∼1.0. The pellet suspension in buffer A containing 20 mM Tris pH 7.5, 500 mM NaCl, 1 mM DTT, and 20 mM imidazole was lysed using the high-pressure cell crusher FB-110X (Shanghai Litu). After centrifuge at 15,000 rpm for 60 min, the supernatant was applied to Ni affinity column (GE Healthcare, 17-5318-02). The target protein eluted from Ni column was cleaved by TEV protease (prepared by our lab) and was then concentrated and applied to Superdex-200 gel-filtration column (GE Healthcare, 28-9909-44) using buffer containing 20 mM Tris pH 7.5, 150 mM NaCl, and 1 mM DTT. The protein after Superdex-200 was rebound to Ni affinity column to remove His-tagged TEV protease and the flowthrough was concentrated into ∼12.5 mg/ml for the purpose of crystallization.

The two double mutants K196E/K197E and R200E/R201E for the lipid strip assay were also purified in a similar way but without the removal of the His-tag. The triple mutant I217M/L251M/L301M, and the double mutants K196E/K197E and R200E/R201E were generated using a similar method described in Stratagene quick-change kit manual. All the mutants were confirmed by DNA sequencing.

For expression of the SeMet-derived Carin2_HR1 triple mutant (I217M/L251M/L301M), we used a method of methionine biosynthesis inhibition as described before (25). Briefly, during the scale-up step, the LB medium is replaced by the M9 minimal media, and the culture was added with 100mg/L phenylalanine, lysine, and threonine, and 50 mg/L leucine, isoleucine, valine, and L-SeMet for 15 min before IPTG induction. The triple mutant was purified similarly as its WT counterpart.

### Crystallization and structure determination

hCap2_HR1 crystals grown from 12 to 15% PEG3350, 20 mM lithiuym chloride belonged to space group C2 with cell parameters a = 139.721 Å; b = 65.323 Å; c = 112.203 Å; α = 90.00°; β = 113.75°; and γ = 90.00°. Crystals were flash-cooled at liquid nitrogen in mother liquid containing 30% glycerol. Diffraction data were collected at Shanghai Synchrotron Radiation Facility Beamline BL17u and processed with the HKL2000 software (https://www.hkl-xray.com/hkl-2000) (26). The crystal structure was determined by Se-MET SAD using Phaser as part of the CCP4 suite (https://www.ccp4.ac.uk/download/#os=windows) (27, 28, 29). Briefly, the experimental phasing searched for four Se sites using the AutoSol run from the PHENIX GUI with the anomalous signal being 0.0711. Then, the model rebuilding was completed using AutoBuild run from the PHENIX GUI with the best solution of R/R_free_ being 0.34/0.37. Refinement steps were performed using REFMAC (30), and model corrections were carried out using Coot (31). Buried surface areas were calculated with PDBePISA. The final R_work_ and R_free_ factors were 0.218 and 0.240. The quality of these models was examined with the program MolProbity (32). The data collection and refinement statistics are presented in Table 1.

**Table 1:** Data collection and refinement statistics

	Native	Se-MET
Data collection		
Space group	*C*2 139.721, 65.323, 112.203 90.00, 113.75, 90.00	
Unit-cell parameters		
*a*, *b*, *c* (Å)		
α, β, γ (°)		
Wavelength	0.9799	0.9792
Resolution range (Å)	50.0–2.32 (2.39–2.32)^a^	50.0–2.58 (2.62–2.58)^a^
Total reflections	231,492	1,091,254
Unique reflections	38,812	29,881
Completeness (%)	97.6 (97.0)	98.8 (99.5%)
Redundancy	6.0 (5.2)	4.7 (5.0)
*R*_merge_	0.124 (0.875)	0.075 (0.806)
*<I*/σ*I>*	31.67 (2.34)	15.01 (2.07)
Refinement		
*R*_work_/*R*_free_	0.218/0.240	
No. of atoms		
Protein	3317	
Solvent	83	
RMSDss		
Bond lengths (Å)	0.004	
Bond angles (°)	0.639	
Ramachandran		
Favored (%)	97.05	
Allowed (%)	2.95	
Outliers (%)	0.00	
Average *B*-factors (Å^2^)	80.43	

a Values in parentheses are for highest resolution shell.

### MALDI-TOF mass spectrometry

After limited trypsinization, the proteolyzed product, after further purified by Superdex-75 gel-filtration column (GE Healthcare, 17-5174-01), was sent to MS analysis along with the freshly prepared protein sample without trypsin treatment. Molecular masses of protein samples were measured on an MALDI TOF-TOF mass spectrometer (AB SCIEX) in a positive ion mode. The system was calibrated immediately before analysis with a mixture of insulin, cytochrome C, apomyoglobin, and mass precision was better than 50 ppm. For MS analysis, a 1 μl volume of the protein solution was mixed with 0.6 μl volumes of solutions of 10 g L^−1^-Sinapic acid matrix prepared in a diluent solution of 50% acetonitrile with 0.1% TFA. The mixture was spotted on a stainless steel Opti-TOF 384 targets; the droplet was allowed to evaporate before introducing the target into the mass spectrometer. A laser intensity of 5500 was typically employed for ionizing. MS spectra were acquired in the positive Liner mode by in the mass range from 20,000 to 100,000 Da. Mass spectra were analyzed with TOF/TOF Series Explorer for TOF software (https://sciex.com).

### Edman sequencing

After SDS-PAGE, the proteolyzed protein was transferred into poly (vinylidene fluoride) membrane in 3-(cyclohexylamino)-1-propane-sulfonic acid buffer. The membrane was stained with ponceau staining solution [0.1% (w/v) ponceau，5% (v/v) acetic acid]. The membrane was air-dried and the protein band was excised from the membrane. Then, the excised band was subjected to amino acid sequencing using an automated protein sequencer PPSQ-33A (Shimadzu), which utilizes phenylisothiocyanate (PITC) reagent to interact with the last N-terminal amino acid residue of the sample protein, forming a PITC-amino acid complex. Under acidic conditions, the PITC-amino acid residue was cleaved and converted to a stable phenylthiohydantoin–-amino acid residue. The PITC reaction was repeated for a total of ten cycles for the identification of the N-terminal amino acid residues of the sample protein.

### Biolayer interferometry assay

Purified hCap_HR1 WT and the double mutant R200E/R201E in buffer X (20 mM Tris pH7.5, 150 mM NaCl, 0.2 mM DTT, 0.05% Tween-20, 0.2 mg/ml bovine serum albumin [BSA]), were concentrated into 25 μM and 23.6 μM, respectively. The biotinylated PI4P (Echelon, C-04B6A) were dissolved in the same buffer. Binding affinities of PI4P to hCap_HR1 WT or R200E/R201E were determined by bio-layer interferometry analysis on an Octet Red96 instrument (Pall FortéBio). In brief, biotinylated PI4P were immobilized onto streptavidin biosensors (Pall FortéBio). After washing with buffer X, these sensors were incubated with 2-fold serial dilutions of sample proteins for 360 s. Subsequently, the biosensors were allowed to dissociate for 360 s. The data were analyzed using the Octet Data Analysis 11.0 software (https://www.sartorius.com) to calculate affinity constants and corresponding errors.

### Lipid strip assay

Membrane lipid strip was bought from Echelon. Purified WT Cap2_HR1 and the mutants were diluted in PBST + 3% BSA with a final concentration of 1.4 μg/ml, which was then incubated with the membrane for 1 h at room temperature in dark. After washing three times with PBST, the membranes were added with anti-His (Sigma, H1029) in PBST + 3% BSA for 1 h at room temperature. After washing three times with PBST, horseradish peroxidase–conjugated goat anti-mouse (Invitrogen, 31430) in PBST + 3% BSA was added for 1 h at room temperature. After washing three times with PBST, the membrane was added with enhanced chemiluminescence substrate (Thermo Fisher Scientific) for 30 s and subjected to exposure by INgenius syngene bioimaging.

### Cell culture and transfection

HEK-293T and U2OS cells (purchased from American Type Culture Collection) were propagated in Dulbecco’s Modified Eagle Medium (Gibco, 12430062) and cultured in 1640 medium (Gibco, 11875093), respectively. Both cell lines were confirmed to be free of *mycoplasma* contamination and authenticated by short tandem repeat DNA profiling. Those cells were grown in culture media supplemented with 10% fetal bovine serum at 37 °C and 5% CO_2_. The appropriate amount of cells (∼1.2 × 10^5^/well) were seeded in 12-well plates 12 h before transfection. Mixture of the target plasmid (Caprin2 WT, R200E/R201E, or K196E/K197E), P3000 and Lipo-3000 (Invitrogen, L3000015) in Opti-minimal essential medium (Gibco, 2193324) was added into cells according to the manufacturer’s instructions of Lipo-3000.

### Viral packaging and infection

The target sequences of shRNAs were reported before, and viral packaging and infection were also carried out as we described previously (17). Briefly, the shRNAs were cloned into the pLKO.1-puro lentiviral vector (Addgene). The target sequences were as follows: 5′-AGCTCAAACTGGAGGATTATA-3’ (shCaprin) and 5′-ACAGTTAACCACTTTTTGAAT-3’ (shCtr). Lentiviral particles were generated by cotransfecting pLKO.1-puro transfer vector, psPAX2, and pDM2.G into HEK293T cells by using Lipo-3000. Forty eight hours later, the supernatant medium was collected and centrifuged at 27,000 rpm for 2 h at 4 °C. The pellet was then resuspended with the Opti-minimal essential medium and added into cells in the presence of polybrane (1:1000). After ∼48 h, the infected cells were used for further related analyses.

### Western blot analysis

Cells were harvested and lysed in 2 × protein loading buffer [0.1 M Tris–HCl pH 6.8, 4% SDS, 2% (v/v) 2-hydroxy-1-ethanethiol, 25% (v/v) glycerol, 0.04% (w/v) bromophenol blue, and proteinase inhibitors] and centrifuged at 5500*g* for 5 min at 4 °C. After separated by SDS-PAGE, proteins were transferred onto a nitrocellulose membrane, and the membrane was then blocked with 5% milk and 2% BSA in Tween-Tris-buffered saline (TTBS) for 1 h at room temperature. After washing three times with TTBS, the membrane was incubated with the primary antibody in TTBS containing 2% BSA at 4 °C overnight. After washing three times with TTBS, the membrane was incubated with the horseradish peroxidase–conjugated secondary antibody at room temperature for 1 h. After washing three times with TTBS, the membrane was added with enhanced chemiluminescence for 10 to 120 s and subjected to exposure by E-blot touch imager (E-blot Xli, E-blot Life Science). Anti-LEF1 (1:1000, Cell Signaling Technology/CST, 2230), anti-Flag (1:1000, CST, 2368), anti-tubulin (1:3000, Sigma, T5168), anti-LRP6 (1:1000, CST, 2560), and anti-phospho-LRP6 (S1490) (1:1000, CST, 2568) were used in this work.

### Immunofluorescence staining

U2OS cells were seeded at 5 × 10^4^/well on glass coverslips (Fisherbrand, 12-545-80) in 24-well plates and allowed to attach overnight. After corresponding plasmids were transfected for 48 h, slides were then fixed with 4% (w/v) paraformaldehyde (Sigma, P6148) in PBS for 10 min at room temperature. After washing with PBS for three times, cells were permeabilized with methanol for 10 min on ice. After washing three times with PBS, cells were blocked with 5% BSA (Sangon Biotech, A500023 CAS: [9048-46-8]) for 1 h. Primary antibodies (anti-Flag: 1:500, CST, 2368; anti-β-catenin: 1:500, BD Biosciences, 610154) were incubated overnight. On the following day, slides were first washed in PBS and then incubated with a secondary antibody (Cy3: 1:1000, Jackson, 115-165-146; Alexa Fluor 488: 1:1000, Molecular Probes, A21206) for 1 h. The slides were counterstained with 4′, 6-diamidino-2-phenylindole at a final concentration of 500 ng/ml (Sigma, D8417) and mounted with antifade mounting medium (Vector, H-1400) for examination. Images were captured by using Leica TCS system (Lecia, Leica TCS SP8).

### Real-time PCR

Total RNAs were extracted using the Trizol Reagent (Invitrogen, 99939401) and reverse transcribed to cDNA using the HiScript III RT SuperMix for qPCR Kit (Vazyme, R323-01), followed by RT-qPCR using the SYBR-Green qPCR Master Mix (Vazyme, Q712), which was performed on the BIO-RAD CFX96 Real-time PCR machine.

## Data availability

Atomic coordinates and diffraction data were deposited at the Protein Data Bank under accession code 8K9C.

## Supporting information

This article contains supporting information.

## Conflicts of interest

The authors declare that they have no conflicts of interest with the contents of this article.

## Supporting information

Supporting Materials

Supporting Figures Caption
