# Supplementary material for: Structural insights into the Caprin-2 HR1 domain in canonical Wnt signaling
Source: J Biol Chem. 2024 Aug 17;300(10):107694. doi: 10.1016/j.jbc.2024.107694 (PMC11480233; doi:10.1016/j.jbc.2024.107694)
Supplement: Supporting Materials [file mmc1.pdf]

Sample Information

Data Filename	: blank_20110314__D01.lcd
Sample Name	: blank
Method Filename	: Manipulation.lcm
Date Acquired	: 2011-3-14 15:42:08
Background Filename	:

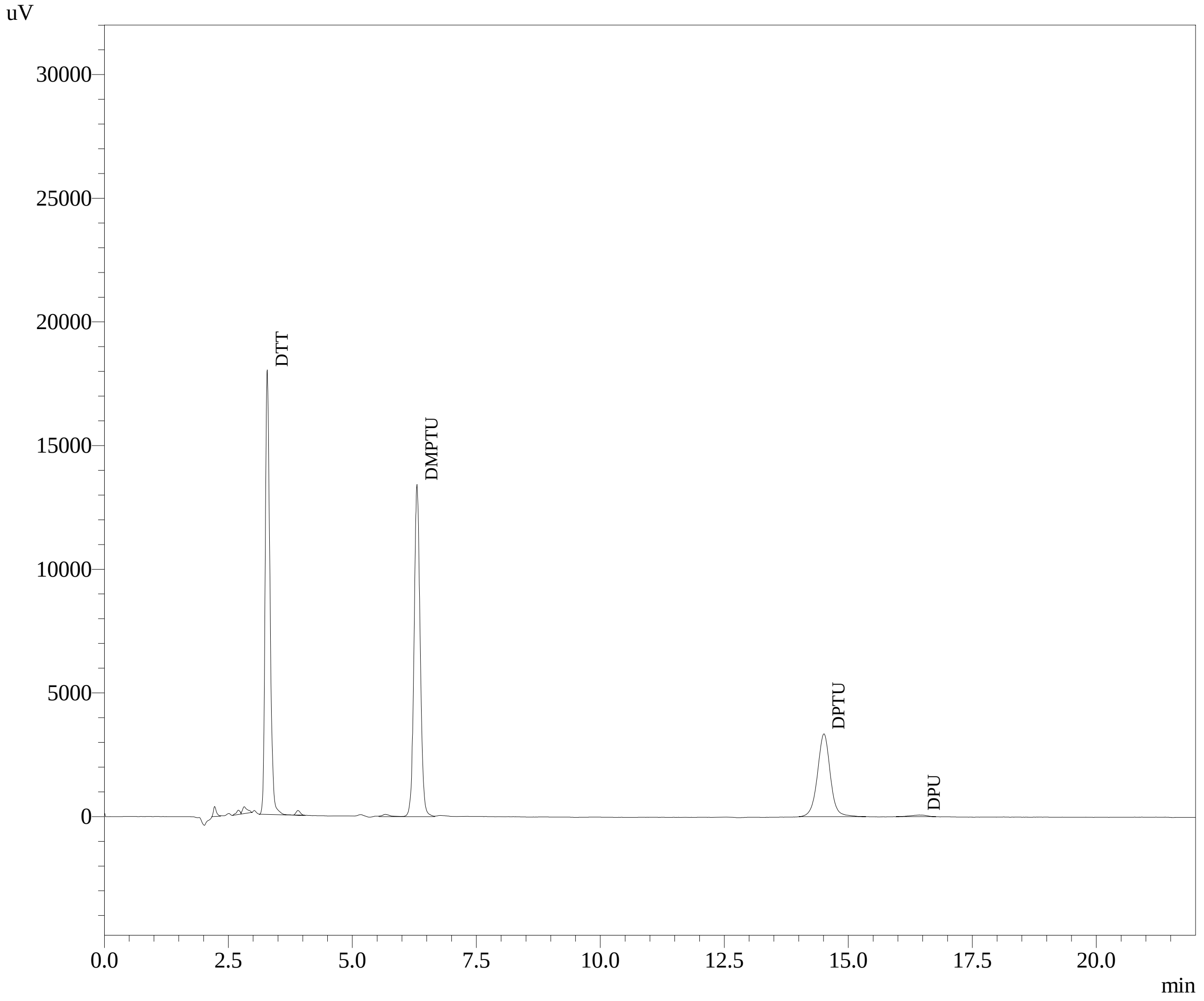

Sample Information

Data Filename : 20110315_PTH-AA_D01.lcd
Sample Name : 20110315_PTH-AA
Method Filename : 20110315_PTH-AA.LCM
Date Acquired : 2011-3-15 9:38:05
Background Filename :

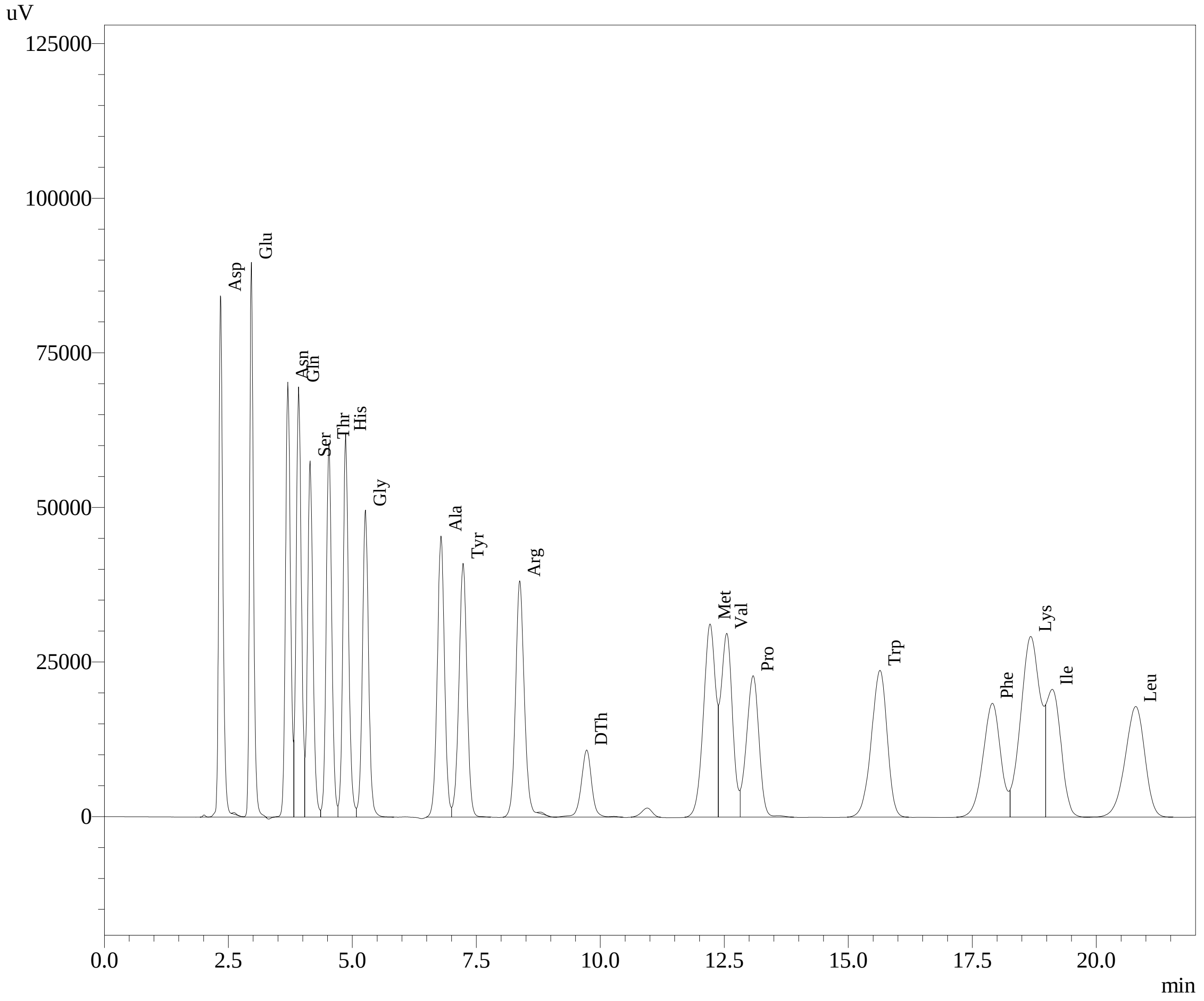

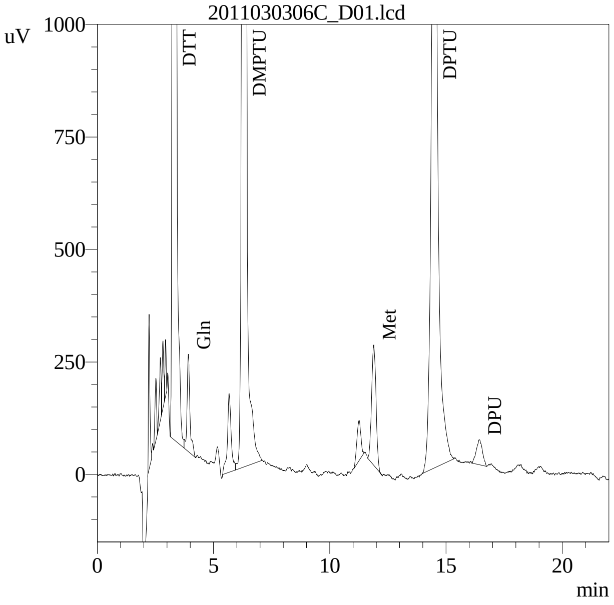
2011030306C_D01.lcd
uV
min
DTT
DMPTU
Gln
Met
DPTU
DPU

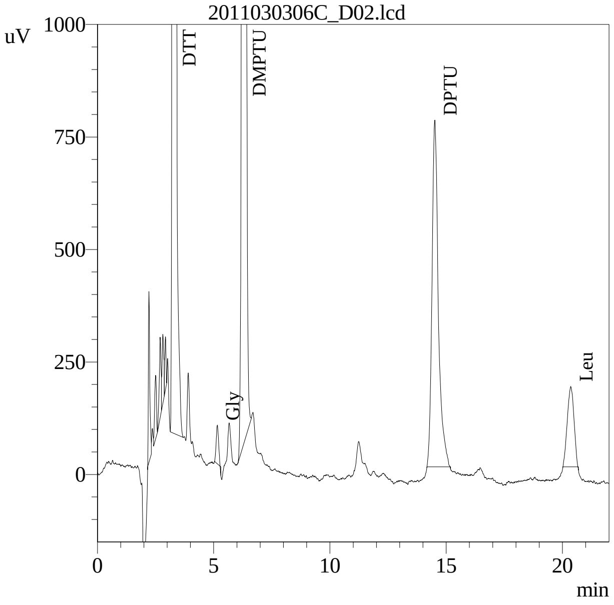
2011030306C_D02.lcd
uV
min
DTT
DMPTU
Gly
DPTU
Leu

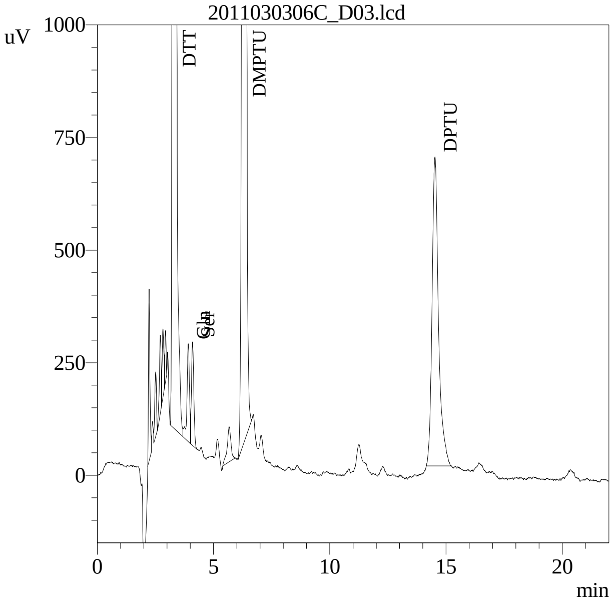
2011030306C_D03.lcd
uV
min
DTT
DMPTU
Gln
Ser
DPTU

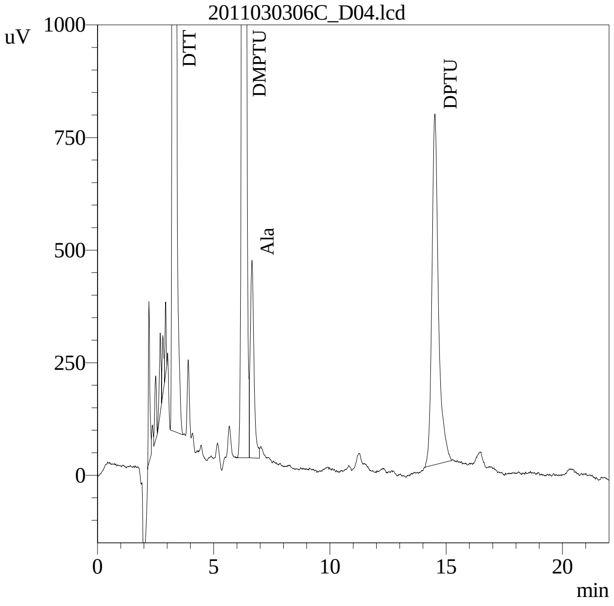
2011030306C_D04.lcd
uV
min
DTT
DMPTU
Ala
DPTU

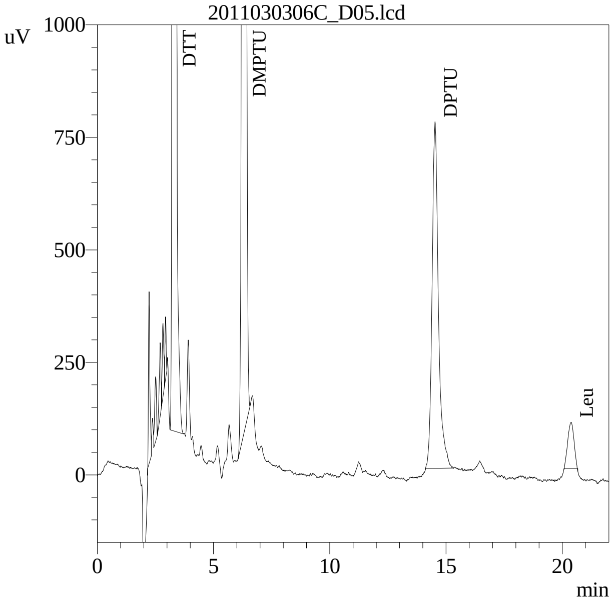
2011030306C_D05.lcd
uV
min
DTT
DMPTU
DPTU
Leu

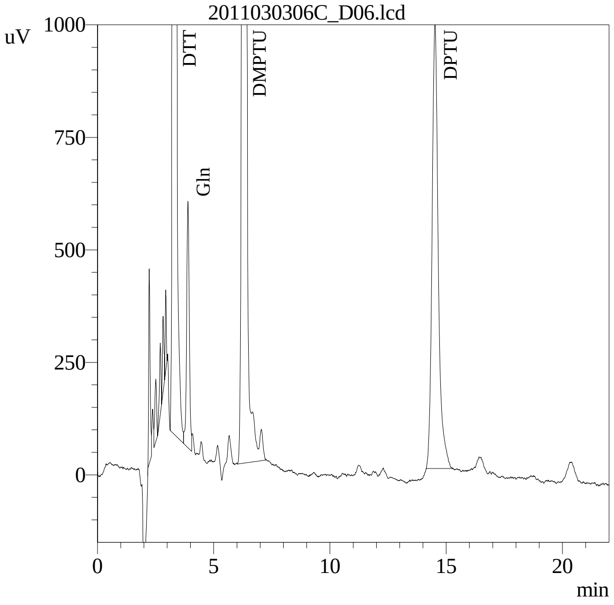
2011030306C_D06.lcd
uV
min
DTT
DMPTU
Gln
DPTU

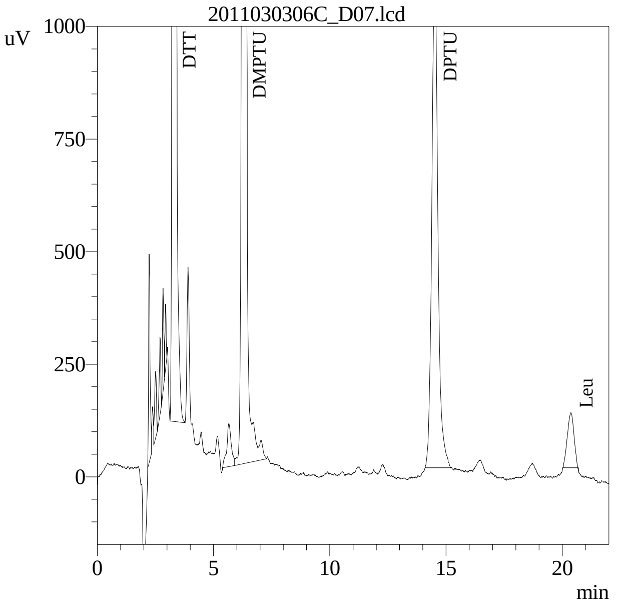
2011030306C_D07.lcd
uV
1000
750
500
250
0
DTT
DMPTU
DPTU
Leu
0
5
10
15
20
min

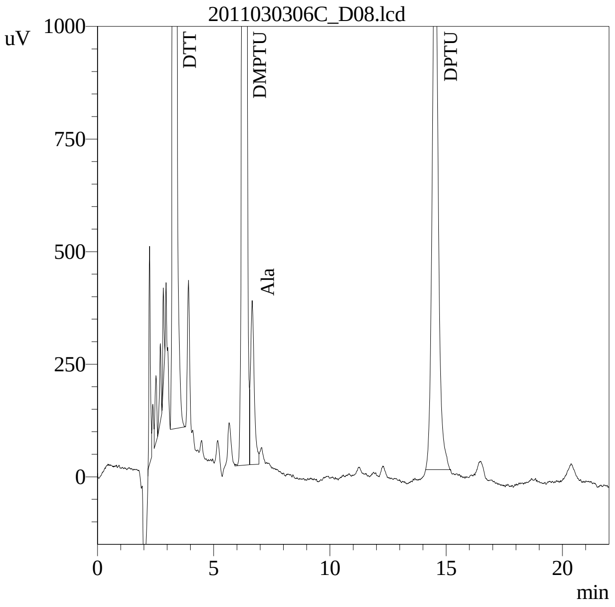
2011030306C_D08.lcd
uV
1000
750
500
250
0
DTT
DMPTU
Ala
DPTU
0
5
10
15
20
min

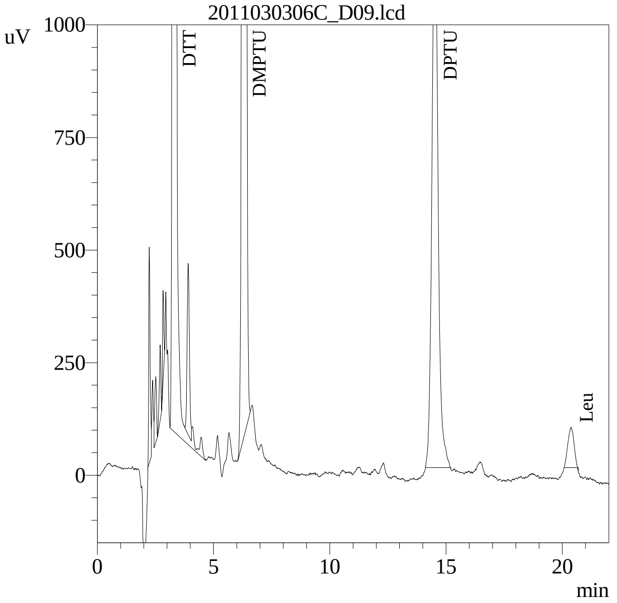
2011030306C_D09.lcd
uV
1000
750
500
250
0
DTT
DMPTU
DPTU
Leu
0
5
10
15
20
min

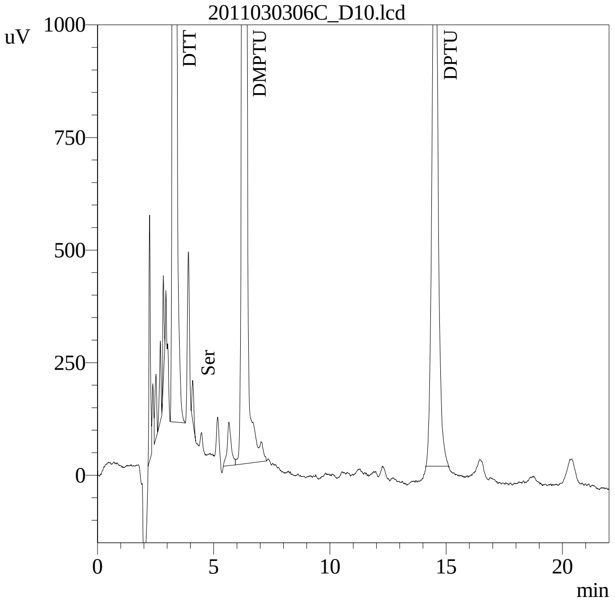
2011030306C_D10.lcd
uV
1000
750
500
250
0
DTT
DMPTU
Ser
DPTU
0
5
10
15
20
min

Final Report

<table>
<tr><td>Sample name</td><td>Cap2-HR1</td><td>Lot number</td><td>--</td></tr>
<tr><td>Sample state</td><td>PVDF</td><td>Estimated MS</td><td>29000 Da</td></tr>
<tr><td>Instrument model</td><td colspan="3">PPSQ-33A
SHIMADZU CORPORATION</td></tr>
<tr><td>Sequencing result</td><td colspan="3">
<table>
<tr><th>Residue No.</th><th>aa name</th><th>Residue No.</th><th>aa name</th></tr>
<tr><td>1</td><td>M、Q</td><td>2</td><td>L、G</td></tr>
<tr><td>3</td><td>S、Q</td><td>4</td><td>A</td></tr>
<tr><td>5</td><td>L</td><td>6</td><td>Q</td></tr>
<tr><td>7</td><td>L</td><td>8</td><td>A</td></tr>
<tr><td>9</td><td>L</td><td>10</td><td>S</td></tr>
</table>
</td></tr>
</table>
