# Supplementary material for: Structural insights into the Caprin-2 HR1 domain in canonical Wnt signaling
Source: J Biol Chem. 2024 Aug 17;300(10):107694. doi: 10.1016/j.jbc.2024.107694 (PMC11480233; doi:10.1016/j.jbc.2024.107694)
Supplement: Supporting Figures Caption [file mmc2.docx]

**FIGURE S1. Determination of the N- and C-terminal boundary of Caprin-2 HR1 domain.** *A*, limited trypsinization of zebrafish Cap2_HR1 (aa S35-D355). Trypsin (SinoPharm) was mixed with zCap2_HR1 protein at different molar ratios as indicated, which was then kept for 10 - 40 min at room temperature. *B*, zebrafish Cap2_HR1 (aa S35-D355) and its proteolyzed product were sent to mass spectrometry analysis for molecular weight determination. *C*, the proteolyzed product of zebrafish Cap2_HR1 was sent to Edman sequencing, which identified the N-terminal 10 residues of the proteolyzed product. *D*, based on the combination of N-terminal amino acid sequencing and mass spectrometry, the N- and C-terminal boundary of zebrafish Caprin-2 HR1 was determined to be M42 and K288 respectively. The N-terminal four extraneous residues (STMD) colored in green result from the introduction of a TEV recognition site as well as the restriction enzyme cloning site. *E*, Sequence alignment of zebrafish and human Caprin-2 suggests that aa A100-K351 of human Caprin-2 could be a compact and stable region that is suitable for structure analysis. Considering four extraneous residues would be introduced into the N-terminus due to the TEV recognition site and restriction enzyme cloning site, S102-K351 of human Caprin-2 was consequently cloned for subsequent studies.

**FIGURE S2. Evaluation of the MW of Caprin-2 HR1 domain in solution.**

*A,* a mixture of five standard proteins (Bio-Rad 1511901) was run on Superdex 200, and the MW and elution peak volume for each standard protein were indicated in the upper left corner. *B,* WT Cap2_HR1 was eluted from the same column with the elution peak being 13.15 ml. *C,* the MW of Cap2_HR1 was calculated to be 66.45 kDa based on the calibration curve generated using the data from *A*. The theoretical MW of Cap2_HR1 based on its primary protein sequence was calculated to be 29.20 kDa.

**FIGURE S3. Verify the knock down efficiency of Caprin-2 by qPCR.**

U2OS cells were infected by lentivirus encoding the shRNA targeting Caprin-2 or by lentivirus encoding the shRNA control for 48 h. Total RNAs were then extracted, followed by qPCR. The relative expression level of mRNA was analyzed by using 2-ΔΔCt method. A 2-tailed T test was used for statistical analysis by using Graphpad. ****P ≤ 0.0001.
